# The effects of diltiazem in renal transplantation patients treated with cyclosporine A^☆^

**DOI:** 10.1016/S1674-8301(10)60044-9

**Published:** 2010-07

**Authors:** Wujun Xue, Yong Song, Puxun Tian, Xiaoming Ding, Xiaoming Pan, Hang Yan, Jun Hou, Xinshun Feng, Heli Xiang, Xiaohui Tian

**Affiliations:** Department of Renal Transplant, Center of Nephropathy, the First Affiliated Hospital of Xi'an Jiaotong University, Xi'an 710061, Shaanxi Province, China

**Keywords:** transplant/kidney, diltiazem, cyclosporine A

## Abstract

**Objective:**

To investigate the effects of diltiazem and cyclosporine A (CsA) combination therapy on protecting the kidney, promoting graft functioning and improving post-transplanted kidney recovery.

**Methods:**

The blood concentrations of CsA, the condition of the post-transplant kidney, the rate of acute rejection (AR), as well as hepatic and renal toxicity in 636 cases of renal transplant recipients were determined after being treated by CsA, with or without diltiazem.

**Results:**

Compared with the control group which received CsA, mycophenolate mofetil (MMF) and prednisolone (Pred) but lacked diltiazem, the group receiving these agents together with diltiazem required reduced dosage of CsA (*P* < 0.01), while blood concentrations of CsA were significantly increased (*P* < 0.01); the recovery time of graft function was reduced from (6.2±1.5) d to (3.9±1.4) d (*P* < 0.01), and the rate of AR was decreased from 13.2% to 7.9% (*P* < 0.01).

**Conclusion:**

In renal transplantation patients treated with CsA and diltiazem, blood concentrations of CsA were increased while the dosage was decreased. This efficient combination therapy reduced patients' economic burden, at the same time retained kidney function, promoted graft function recovery and decreased hepatic and renal toxicity and the rate of AR.

## INTRODUCTION

Cyclosporine A (CsA), a calcineurin inhibitor, has become the most important immunosuppressive medication used widely by patients who receive solid organ transplants since its introduction to clinical practice in 1978. However, because of its high price, narrow therapeutic index and inter-individual variability, close drug monitoring programs have to be undertaken for its clinical application[1],[2]. In clinical practice, therapeutic drug monitoring for immunosuppressive agents is routinely performed in order to optimize the efficacy and limit the medication toxicity[3],[4].

CsA is frequently co-administered with diltiazem, because the latter has possible beneficial effects on the economic impact associated with reduction of the dose of CsA[5]. Diltiazem is a relatively safe drug with useful antihypertensive effects on the control of blood pressure and protection of kidney function[6]. However, the diltiazem group is associated with a significantly higher probability of having chronic allograft nephropathy than the non-diltiazem group[7].

Some studies have shown that there were inter-individual and inter-ethnic differences in CsA pharmacokinetics[8]. For renal transplant patients, achieving target blood concentrations of CsA as soon as possible after transplantation is the key to preventing rejection[9]. There is no published report that mycophenolate mofetil (MMF) combined with diltiazem and CsA affected the CsA concentration fluctuation after renal transplantation in the northwest of China, especially in the 12 y follow-up of clinical studies in our center. In order to correct such a situation, we have conducted a series of experiments[10]. The purpose of this study is to assess the influences of diltiazem on the blood concentrations and pharmacokinetics of CsA in kidney transplant recipients.

## MATERIALS AND METHODS

### Subjects

Two thousand three hundred and fifteen patients received renal transplantation in our hospital from January 1987 to December 2009, and 1,692 of them were treated with an immunodepressive program of CsA only, while 872 patients received CsA, MMF and prednisolone (Pred) for one year. Since 1990 we have found that the combination therapy of diltiazem and CsA can obtained a better clinical effect, and the detailed report is as follows.

Within 1 y after allograft renal transplantation, 872 recipients were randomly divided into two groups: treatment group (636 patients) and control group (184 patients), and 52 patients quitted the study because of changes in their immunosuppressive medication. The data for age, sex, body weight and body mass index (BMI) were comparable in the two groups (***Table 1***).

**Table 1:** Demographic data of two groups of renal transplantation in our hospital from 1987 to 2009

Characteristics	Treatment group (*n =* 636)	Control group (*n =* 184)	*P*
Age (y)	41.5 ± 10.3	39.3 ± 10.9	0.61
Sex (male/female)	474/162	137/47	0.53
Body weight (kg)	52.8 ± 12.2	55.9± 9.3	0.65
Body mass index (kg/m^2^)	21.5 ± 3.1	21.9± 3.4	0.52

(mean±SD)

The study was approved by the Ethics Committee of the First Affiliated Hospital of Xi'an Jiaotong University and performed in compliance with the Declaration of Helsinki. All volunteers gave written informed consents and the ethics committee of the First Affiliated Hospital of Xi'an Jiaotong University approved the protocol before the trial started.

### General data

There were 820 patients enrolled into our study from 2,315 renal transplantation recipients. Among them, 611 cases were male, and 209 cases were female. The age range was 15-73 y and the average age was (37.2±4.0) y. There were 605 cadaveric renal transplantation cases, and the donor's age range was 18-59 y, and the average age was (23.6±3.0) y. Those patients who were less than 40 years old accounted for 95.3%. Warm ischemia time was (5.0±2.0) min, and cold ischemia time was (6.0±2.0) h. There were 215 living-related donor kidney transplantation (LDKT) cases, with warm ischemia time (4.0±0.5) min, cold ischemia time (10.0±6.0) min.

### Tissue matching

Human leukocyte antigen (HLA) -A, B, DR matching: 14 cases were six antigen mismatches; 78 cases were five antigen mismatches; 163 cases were four antigen mismatches; 206 cases were three antigen mismatches; 351 cases were two antigen mismatches; 8 cases were zero antigen mismatch; crossed matches were all negative (<10%). Detected panel reactive antibody (PRA) was performed after 1997, and 752 cases were <10%; 67 cases were 11%-49%; 1 case was >50%. ABO blood type between donors and recipients accorded with transfusion principle. The different matches groups equally represented between the control and treatment groups.

### Immunosuppressive treatment

#### Treatment group (with diltiazem)

Since January 1997, there were 636 cases treated with the CsA+MMF+Pred combined with diltiazem. The initial dose of CsA was 4.5-5.0 mg/(kg·d); MMF was taken orally at the first day after the transplant operation at a dose of 1.5-2.0 g/d (above 60 kg take 2.0 g/d); CsA and Pred were administered synchronously together with oral co-administration of diltiazem. The initial dose of Pred was 40 mg/d, and the oral maintenance dose of both drugs given together was 15-20 mg/d. In general, the capsule of CsA was made by Sandimmun Novartis pharmaceutical company, China.

Diltiazem was taken orally at the first day after transplant operation at the dose of 60 mg (t.i.d.). Patients in this study did not take any medication known to affect CsA pharmacokinetics, including CYP3A inducers or inhibitors, such as antiepileptics (phenytoin and carbamazepine), antimycotics (fluconazole and ketoconazole), macrolide antibiotics (erythromycin and clarithromycin) and St. John wort.

#### Control group (without diltiazem)

The patients in the control group received starch placebo pills in the single blind study. There were 184 cases treated with CsA+MMF+Pred as control.

### Monitoring during the post-transplantation period

Venous blood samples for morning trough steady blood concentrations of CsA and biochemistry indexes were taken at the beginning and in the end of the study over a period of 12 month (final visit). After the first measurement of blood concentrations of CsA, the patients in the treatment group took diltiazem tablets orally (60 mg, t.i.d.) for 1 y. Blood concentrations of CsA were analyzed by fluorescence polarization immunoassay with TDx (i1000, Abbott Laboratories, USA) and monoclonal antibody kit (Dade Behring Company, USA). Serum total bilirubin (TB), alanine aminotransferase (ALT), albumin (A), blood urea nitrogen (BUN) and creatinine (Cr) were measured using an automatic biochemistry analyzer (7170, Hitachi Company, Japan). Fluorescent polarization immunoassay (FPIA) was used to detect and monitor the Blood trough concentrations of CsA. The other monitored targets included routine blood and urine tests, hepatic and renal function tests, urinary fall-off cells, isotope kidney dynamic, multicolor ultrasonic, transplant kidney biopsies and so on.

### Statistical analysis

All data are expressed as mean±standard deviation (SD). The comparison of sample average values was run by t test, and the comparison of frequency in two different groups was performed using the chi-square test. Statistical analyses were carried out using SPSS software 10.0 (SPSS Inc., USA). *P* < 0.05 was considered statistically significant.

## RESULTS

### Comparison of hepatic and renal fundtion of patients in two groups

Measurements of hepatic and renal functions are shown in ***Table 2***. In the treatment group, the albumin levels at the final visit were significantly higher than those in the control group (*P* < 0.01), but the values were still within the normal range. The mean Cr level in the control group was higher than that in the treatment group (*P* < 0.05), but the mean Cr levels in both groups were reduced to almost the normal range by the end of this study. No significant difference in TB, ALT, BUN or creatinine clearance (CLCr) was revealed (*P* > 0.05) between the two groups. During the study, fourteen patients had short periods of hypotension, but no other adverse effects were observed.

**Table 2:** Comparison of the post-renal transplantation hepatic and renal function of two groups

Group	Treatment time				
	1 month	3 month	6 month	9 month	1 y
Treatment (*n =* 636)					
Total bilirubin (µmol/L)	10.90 ± 5.10	12.70 ± 6.30	14.50 ± 4.70	13.80 ± 3.90	13.20 ± 4.20
ALT (U/L)	36.30 ± 23.10	34.30 ± 22.70	34.60 ± 14.70	29.80 ± 9.20	23.30 ± 12.70
Albumin (g/L)	38.10 ± 4.30**	44.20 ± 5.10**	47.20 ± 3.50**	43.80 ± 5.20*	41.60 ± 3.70*
BUN (mmol/L)	12.10 ± 4.90	10.40 ± 5.70	11.20 ± 6.10	10.90 ± 5.80	11.90 ± 4.30
Cr (µmol/L)	127.30 ± 24.30*	116.30 ± 32.10*	101.70 ± 27.90*	91.80 ± 24.50*	81.30 ± 21.90*
CLCr (mL/min)	73.80 ± 17.30	55.10 ± 16.20	53.80 ± 17.60	73.80 ± 17.30	73.80 ± 17.30
Control (*n* = 184)					
Total bilirubin (µmol/L)	12.30 ± 3.70	14.50 ± 4.20	15.30 ± 4.70	16.10 ± 3.20	15.40 ± 3.70
ALT (U/L)	38.30 ± 28.60	32.60 ± 21.30	38.40 ± 17.50	32.80 ± 13.70	25.30 ± 14.30
Albumin (g/L)	31.20 ± 5.40	36.50 ± 4.30	38.10 ± 3.70	38.40 ± 4.60	38.30 ± 3.40
BUN (mmol/L)	13.60 ± 3.80	11.60 ± 4.40	12.10 ± 3.90	11.20 ± 4.30	11.20 ± 3.20
Cr (µmol/L)	134.70 ± 37.10	123.10 ± 29.40	118.40 ± 23.30	97.10 ± 27.70	89.50 ± 26.40
CLCr (mL/min)	77.20 ± 16.80	58.00 ± 17.30	55.60 ± 15.20	75.20 ± 15.60	76.20 ± 16.70

*P < 0.05 *vs* control group of the same parameter at the same time period; **P < 0.01 *vs* control group of the same parameter at the same time period.

(mean±SD)

### The dosage and blood concentrations of CsA at different post-transplantation times

The dosage of CsA in the treatment group was less than that used in the control group (*P* < 0.01), and the blood concentrations of CsA were higher in the treatment group than in the control group (*P* < 0.01). There was also significant difference between treatment group and control group in the ratio of blood concentration of CsA to dose of CsA (***Table 3***), especially 6 and 9 months after the transplantation (*P* < 0.01).

**Table 3:** The difference between treatment group and control group CsA dosage and blood concentrations

Group	Treatment time				
	1 month	3 month	6 month	9 month	1 y
Treatment (*n =* 636)					
CsA [(mg/(kg·d)]	4.26 ± 0.24*	3.82 ± 0.33*	3.25 ± 0.28*	2.96 ± 0.23*	2.51 ± 0.29*
CsA dosage (mg/d)	234.30 ± 72.10**	207.30 ± 62.70**	188.60 ± 58.70**	162.80 ± 68.20**	143.10 ± 58.70**
CSA concentration (Hg/L)	245.60 ± 53.70**	221.80 ± 42.50**	204.70 ± 31.40**	179.20 ± 43.90**	152.60 ± 36.40**
Concentration/dose	1.17 ± 0.79*	1.09 ± 0.69*	1.10 ± 0.57**	1.13 ± 0.68**	1.08 ± 0.67*
Control (*n =* 184)					
CsA [(mg/(kg·d)]	4.56 ± 0.26	4.12 ± 0.23	3.75 ± 0.24	3.39 ± 0.21	3.01 ± 0.31
CsA dosage (mg/d)	287.40 ± 63.50	251.70 ± 69.40	216.30 ± 61.80	188.60 ± 59.70	167.50 ± 60.30
CSA concentration (Hg/L)	220.30 ± 45.60	190.10 ± 41.40	153.00 ± 37.30	125.30 ± 32.50	109.80 ± 27.10
Concentration/dose	0.81 ± 0.72	0.80 ± 0.61	0.74 ± 0.59	0.75 ± 0.54	0.70 ± 0.49

*P < 0.05 *vs* control group; **P < 0.01 *vs* control group.

### The outcome of transplantation

**Fig. 1:**
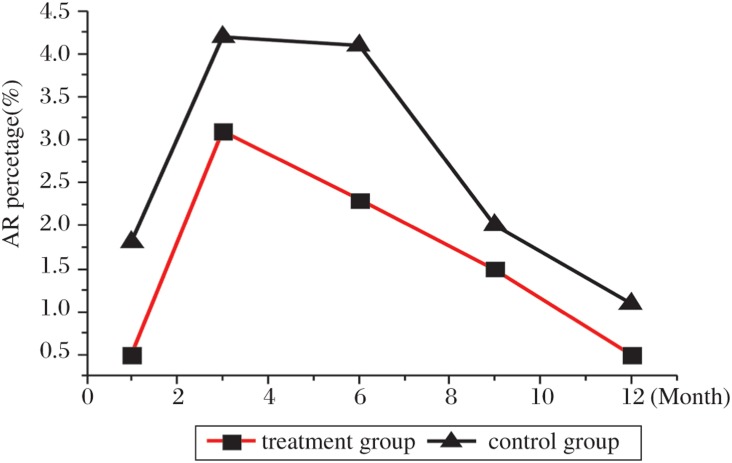
Comparison of the ratio of AR between the treatment group and control group. The rate of AR in the treatment group was significantly lower than that in the control group, especially at month 3 and 6.

At 1, 3 and 5 y, the patient/graft survival rate (%) in the treatment group was 95.4/93.6, 83.8/73.8 and 69.9/65.1 respectively; whereas in the control group, it was 87.3/85.7, 69.5/62.7 and 61.2/57.9. There were 50 cases of AR (the rate was 7.9%) in the treatment group, and the rate was significantly lower than that in the control group (13.2%, *P* < 0.01). The difference was larger between month 3 and 6 (***Fig. 1***). The primary post-transplantation complication of the two groups is shown in ***Table 4***. In the treatment group, both CsA hepatic toxicity (%) and CsA renal toxicity (%) were significantly less than those in the control group (*P* < 0.01), but the infection rate was slightly more than that in the control group (*P* < 0.05).

**Table 4:** The comparison of complications between the treatment group and control group

Complications	Treatment group [*n* (%)]	Control group [*n* (%)]	*P*
CsA hepatic toxicity	78 (12.3)	28 (15.4)	0.008
CsA renal toxicity	42 (6.7)	23 (12.6)	0.004
Infection	224 (35.3)	60 (32.7)	0.041

### The recovery time for graft kidney

The recovery time to normal function of the grafted kidney after the transplantation was (6.2±1.5) d and (3.9±1.4) d in the control group and treatment group, respectively. The difference between treatment group and control group was statistically significant (*P* < 0.01).

## DISCUSSION

The outcome of kidney transplantation has become much better since the introduction of CsA. However, CsA is relatively expensive in China, resulting in a heavy economic burden to patients treated orally with triple therapy that includes CsA. Many patients have to stop or reduce the dosage of CsA without the permission of their doctor, especially in the poorer area (northwest) of China. This raises the incidence of rejection, and greatly affects the long-term graft survival and life quality of the patients. Therefore, it is important to consider how to ensure a better outcome of kidney transplantation while reducing the cost, especially medication costs. In 1986, Griño *et al*[11] first reported that the combination of diltiazem increased blood concentrations of CsA and reduced the required dosage of CsA. Since 1988, in order to increase CsA blood concentration, reduce CsA dosage and decrease medication cost[12],[13], we have combined diltiazem with CsA in renal transplant patients. A better treatment outcome was confirmed by the 636 cases in our clinical study.

The possible mechanism by which diltiazem raises CsA blood concentrations is that diltiazem inhibits the activity of cytochrome P450 enzymes, which are the key enzymes participating the metabolism of CsA[13],[14] in hepatic microsomes[9],[14],[15],[16]. In our study, the dosage of CsA in the treatment group was reduced by more than 15% of the control group (P < 0.01), but blood concentrations were higher than those in the control group (P < 0.01). Due to reducing the dosage of CsA, co-administration of diltiazem could reduce the economic burden on patients, ensure a better transplantation outcome and offer an effective method for the long-term administration of CsA[17],[18]. McCauley et al[19] reported that the daily dosage of CsA was reduced by 30%–50% when combined with diltiazem, and this could save about $3,000/y[19],[20]. Our research showed that the required CsA dosage was reduced by 10%–15% when combined with diltiazem in comparison with the control group, saving about Ұ10,000/y. Owing to the reduced dosage of CsA, the incidence of hepatic and renal toxicity was distinctly reduced, and thus the cost of treating hepatic and renal complication was also decreased[21],[22], so the total expenditure in kidney transplantation was further reduced.

Besides raising CsA blood concentrations, the diltiazem combination also protects renal function by defending against direct renal cytotoxicity and the hemodynamic turbulence caused by CsA. The possible mechanisms include: ① Antagonizing the contstrictive effect on afferent glomerular arteriole caused by CsA, depressing the resistance of renal blood vessel, increasing blood flow, and enhancing glomerular filtration[23]. ② Restraining the feedback of glomerulusrenal tubules, inhibiting the contractile effect of mesangial cells and the glomerular filtration caused by CsA. ③ Abatement of Ca^2+^ inward current and Ca^2+^ channels activation caused by CsA, and blockage of renal toxicity caused by Ca^2+^ dependent reaction[24]. ④ Increasing the conversion of CsA to M17 and other metabolites. The immunosuppressive effect of M17 was same as that of CsA, and its renal toxicity was significant less[16],[25],[26].

Due to the above effects and characteristics of diltiazem, oral treatment with diltiazem improves the outcome of transplantation[27],[28],[29]. In our study, the albumin levels at the final visit of patients in the treatment group were significantly higher than those in the control groups (***Table 4***, P < 0.01), but the values were still within the normal range. It appeared to be safe because most values for hepatic and renal functions were better than those in the control group, for example, Cr levels in the control group was higher than that in the treatment group (P < 0.05). Moreover, no chronic rejection occurred in these patients during this trial. At the end of this study, the patients in the treatment group required reduced dosages of CsA to maintain blood concentrations in the therapeutic window. When MMF was administered, the immunosuppressive effect was boosted, and the rate of AR significantly decreased from 13.2% to 7.9%, and hepatic and renal toxicity were dropped to 12.3% and 6.7% respectively. All these decreases were statistically significant (P < 0.01). These results suggested that CsA+MMF+Pred co-administered with diltiazem was an important way to reduce the incidence of rejection and the damage of hepatic and renal toxicity. However, the incidence of infection was increased slightly, though not significantly, in this treatment group, presumeably due to the immunosuppressive effect that was reinforced by the CsA concentrection affected by diltiazem.

Owing to the facts that diltiazem reduces inward Ca^2+^ current, blocks phosphodiesterase activity and the conversion from xanthine dehydrogenase to xanthine oxidase, decreases oxygen free radicals, and mitigats reactive oxygen species damage, this agent accelerated graft recovery. Thus CsA co-admission with diltiazem early after transplantation could improve the graft function and provide benefit for the long-term survival of the graft[30],[31]. In our study, the graft recovery time in the treatment group was shortened to 3.9 d (*P* < 0.01), suggesting that the diltiazem combination improved graft function.

To the best of our knowledge, this is the first study to examine the interaction between two immunosuppression plans that includes CsA and diltiazem in a large scale clinical study. The results of our study demonstrate that diltiazem administrated together with CsA could enhance blood CsA levels. The results further confirm that an interaction between diltiazem and CsA does exist. In addition, diltiazem appears to be safe because it improves hepatic and renal functions. Moreover, diltiazem is easy to be obtained and inexpensive. It is suitable for the kidney transplant recipients in the northwest of China to reduce treatment costs.

In conclusion, co-administering diltiazem and CsA capsules as an immunosuppressive therapy in kidney transplantation could increase CsA blood concentration, reduce the dosage of CsA, and further lighten patients' economic burden. At the same time, it could also protect kidney function, promote graft function recover, decrease the incidence of AR and kidney toxicity and improve the life quality of kidney transplantation recipients. Nonetheless, administered of diltiazem combined with triple immunodepression therapy including CsA may need further studies to individualize the initial diltiazem dose and immunosuppressive agents such as tacrolimus in transplant recipients during the post-transplant period.
